# Comparative study on three viral enrichment approaches based on RNA extraction for plant virus/viroid detection using high-throughput sequencing

**DOI:** 10.1371/journal.pone.0237951

**Published:** 2020-08-25

**Authors:** Yahya Zakaria Abdou Gaafar, Heiko Ziebell

**Affiliations:** Julius Kühn Institute (JKI)–Federal Research Centre for Cultivated Plants, Institute for Epidemiology and Pathogen Diagnostics, Braunschweig, Germany; Washington State University, UNITED STATES

## Abstract

High-throughput sequencing (HTS) has become increasingly popular as virus diagnostic tool. It has been used to detect and identify plant viruses and viroids in different types of matrices and tissues. A viral sequence enrichment method prior to HTS is required to increase the viral reads in the generated data to ease the bioinformatic analysis of generated sequences. In this study, we compared the sensitivity of three viral enrichment approaches, i.e. double stranded RNA (dsRNA), ribosomal RNA depleted total RNA (ribo-depleted totRNA) and small RNA (sRNA) for plant virus/viroid detection, followed by sequencing on MiSeq and NextSeq Illumina platforms. The three viral enrichment approaches used here enabled the detection of all viruses/viroid used in this study. When the data was normalised, the recovered viral/viroid nucleotides and depths were depending on the viral genome and the enrichment method used. Both dsRNA and ribo-depleted totRNA approaches detected a divergent strain of Wuhan aphid virus 2 that was not expected in this sample. Additionally, Vicia cryptic virus was detected in the data of dsRNA and sRNA approaches only. The results suggest that dsRNA enrichment has the highest potential to detect and identify plant viruses and viroids. The dsRNA approach used here detected all viruses/viroid, consumed less time, was lower in cost, and required less starting material. Therefore, this approach appears to be suitable for diagnostics laboratories.

## Introduction

Viruses and viroids are one of the major emerging threats to agricultural and horticultural production [1]. Climate change and increasing global trade are only two of many factors accelerating the dispersal of plant viruses into new geographic areas where they can potentially cause greater damage thus threating food supplying for humans and animals [2–5]. Precise identification of the virus causing the disease is important for correct management procedures, such as switching to virus-resistant cultivars (where available), quarantine or eradication measures or vector control. In the past, traditional detection methods such as serological (i.e. ELISA, Tissue blot-ELISA) or nucleic acid-based (PCR, probe-based methods) detection methods required a *priori* knowledge of the pathogen that needed to be detected, such as previously purified virus particles used for raising antibodies or nucleic acid sequences for the design of specific primers or target probes [6]. However, divergent sequences or virus variants with different antigenic epitopes on the virion surface would not be detected using these methods. Electron microscopy is important for viruses’ detection, as it requires no prior knowledge for virus detection [7,8]. Nevertheless, it tends not to be able to identify viruses up to species level as its detection is based on the particle morphology, and often fails to detect low titre viruses or viruses that are phloem-limited and disease-causing entities without protein shells such as viroids or satellite RNAs [9,8,10,11].

The development and evolution of novel high-throughput sequencing (HTS) technologies has revolutionised virus discovery, plant virus diagnostics as well as metagenomic, evolutionary and community studies in recent years [12,13]. No prior knowledge about the pathogen is needed for HTS since all the nucleic acid (viral or non-viral) in the sample can be sequenced. Many previously undescribed viruses and viroids have been discovered using HTS and the number is constantly increasing [14–17]. However, HTS-based approaches for virus/viroid detection is facing several challenges in order to be validated for routine use diagnostic laboratories [18]. As HTS can sequence all nucleic acids within a given sample, whether of plant or pathogen origin, suitable enrichment strategies should be used to minimise the generated host reads that reduces the pathogen reads, and may interfere with subsequent bioinformatic analyses. Additionally, there is no universal sequence that can be used for the analysis of virus/viroid communities in contrast to fungi or bacteria where the internal transcribed spacer (ITS) or the 16S ribosomal RNA can be used in amplicon sequencing manner for the general detection of these pathogens [19]. Thus, in case of unknown virus/viroid infections, studies rely on untargeted identification approaches utilizing random primers for detection [20].

As viruses have different genome types, i.e. single- or double-stranded DNA, single- (positive [+ve] or negative [-ve] sense) or double-stranded RNA (dsRNA), circular or non-circular nucleic acids, there are various extraction methods and enrichment strategies available that may or may not suit all molecules equally [8,21]. For example, the extraction of dsRNA (the replicative form of most plant viruses) has been used for a long time to generate sequence information from unknown plant viruses and can be used for the enrichment of viral sequences for HTS [22–25]. More recently, virus-derived small RNAs (vsRNAs) or ribo-depleted total RNA extracts have been used to prepare samples for HTS [26,27]. Alternatively, rolling circle amplification and subsequent sequencing worked well for viruses with circular DNA genomes [28,29]. Viroids, in contrast to viruses, consist of small, circular, single-stranded, non-coding RNA molecules that can fold into secondary structures [30,31]. Viroids replicate via RNA-based rolling circle mechanism in symmetric and asymmetric pathways depending on the viroid family [32]. Few studies have directly compared different enrichment strategies and their ability to detect plant virus sequences through HTS [33,34]. In this study, we compare three RNA based-enrichment strategies, i.e. dsRNA extraction, ribo-depleted totRNA and small RNA (sRNA) extraction, and evaluate their potential for virus/viroid detection, their genome coverage recovery rate and depth recovery from the reads. We included viruses with different genomes ((+ve) ssRNA, (-ve) ssRNA and ssDNA) as well as a viroid.

## Material and methods

### Plant cultivation

Four plant varieties (*Nicotiana benthamiana* [cultivar: JKI-Wild], *Pisum sativum* [cultivar: Rainier], *Solanum lycopersicum* [cultivar: Linda] and *Vicia faba* [cultivar: Tattoo]) were used (Table 1). In addition, *Phaseolus vulgaris* (cultivar: Black Turtle) infected with the cryptic viruses Phaseolus vulgaris alphaendornavirus 1 and 2 (PvEV1 and PvEV2; JKI ID 31403) was used to spike the samples during extraction as control (kindly provided by Dr. Mike Rott). The plants were kept under greenhouse conditions (at 22°C; photoperiod of 16 h light [natural daylight with additional growth light Phillips IP65, 400 Watt] and 8 h dark).

**Table 1:** List of plant viruses and the viroid used in this research.

Sample	Name	Acronym	JKI ID number	Genome nature			Family	Genus	Host
				Type	Organization	Size (bp)			
1	**pea enation mosaic virus 1**	PEMV1	31399	+ssRNA	Linear/ Non-segmented	5,706	*Luteoviridae*	*Enamovirus*	*Pisum sativum*
2	**pea necrotic yellow dwarf virus**	PNYDV	31400	ssDNA	Circular/ segmented	7,896	*Nanoviridae*	*Nanovirus*	*Vicia faba*
3	**Physostegia chlorotic mottle virus**	PhCMoV	31401	-ssRNA	Linear/ Non-segmented	13,321	*Rhabdoviridae*	*Nucleorhabdovirus*	*Nicotiana benthamiana*
4	**potato spindle tuber viroid**	PSTVd	31402	viroid	Circular/ Non-segmented	360	*Pospiviroidae*	*Pospiviroid*	*Solanum lycopersicum*
Internal controls	**Phaseolus vulgaris alphaendornavirus 1**	PvEV1	31403	dsRNA	Linear/ Non-segmented	~14,072	*Endornaviridae*	*Alphaendornavirus*	*Phaseolus vulgaris*
	**Phaseolus vulgaris alphaendornavirus 2**	PvEV2		dsRNA	Linear/ Non-segmented	~14,817	*Endornaviridae*	*Alphaendornavirus*	*Phaseolus vulgaris*

### Viruses and viroid isolates

Three viruses with different genomes and one viroid were used in this study (Table 1). Pea enation mosaic virus 1 (PEMV1), originally from a *P*. *sativum* plant showing enation symptoms, was collected in 2011 from Hondeghem, northern France. Pea necrotic yellow dwarf virus (PNYDV), Elbtal isolate, originally from an infected *P*. *sativum* sample showing dwarfing and yellowing in top leaves and leaf rolling symptoms, originally from Saxony, Germany in 2011. The original Physostegia chlorotic mottle virus (PhCMoV), HZ16-558 isolate, was from an infected *S*. *lycopersicum* plant, collected from Hesse state in Germany displaying fruit marbling and discoloration symptoms in 2016 [20]. The potato spindle tuber viroid (PSTVd), isolate PV-0950, was kindly provided by DSMZ (German collection of microorganisms and cell cultures, Braunschweig, Germany) in the form of lyophilized infected *S*. *lycopersicum* plant leaves in 2014.

### Virus maintenance

PEMV1 and PNYDV were maintained by aphid transmission using *Acyrthosiphon pisum*. The aphids were reared for five days on infected *P*. *sativum* and *V*. *faba*, respectively, and ten viruliferous aphids were transferred onto healthy plants. The inoculation access period was five days. The aphids were killed using a non-systemic insecticide (Spruzit Schädlingsfrei, Neudorff GmbH KG, Emmerthal, Germany).

PhCMoV and PSTVd were maintained by mechanical transmission to *N*. *benthamiana* and *S*. *lycopersicum*, respectively. For mechanical transmission, 100 mg of infected plant material were ground in Norit buffer (0.05M phosphate buffer [pH 7.0]; 0.001M ethylenediaminetetraacetic acid; 0.02M sodium diethyldithiocarbamic acid; 0.005M thioglycolic acid; 0.75% activated charcoal [Norit]) and 30 mg of diatomaceous earth (Celite) were added. The homogenate was rubbed gently on healthy plants’ leaves using glass rods. The inoculated leaves were rinsed with water within 5 min. After inoculation, all plants were kept under greenhouse conditions for four weeks until virus symptoms were observed (except for PSTVd; no symptoms were observed).

### Confirmation of infection by DAS-ELISA and/or PCR/RT-PCR

To confirm infection, all plants were tested by ELISA and/or RT-PCR or PCR (Table 2). ELISA tests were performed (except for PSTVd infected plants) using antibodies listed in Table 2 as described in [35,36]. Additionally, to confirm infections with PEMV1, PhCMoV and PSTVd, RT-PCR was performed using total RNA extracted with innuPREP Plant RNA Kit (Analytik Jena, Jena, Germany), following the manufacturer’s instructions. cDNA was synthesised using ProtoScript II Reverse Transcriptase (New England Biolabs, Frankfurt am Main, Germany) using the reverse primer of the primer pairs mentioned for each virus (Table 2). PCR was performed using OneTaq DNA Polymerase (New England Biolabs, Frankfurt am Main, Germany) and each virus specific primers (Table 2). PNYDV infection was confirmed by DNA extraction according to Edward’s method for plant DNA extraction with 0.1% Mercaptoethanol added to the extraction buffer [37]. The extracted DNA was treated by RNase A (Carl Roth, Karlsruhe, Germany), followed by PCR using OneTaq DNA Polymerase (New England Biolabs, Frankfurt am Main, Germany) and PNYDV specific primers

**Table 2:** Virus/viroid infection confirmation. Symptoms, ELISA and RT-PCR or PCR including antibodies and primer sets used for confirmation, and the reference sequences used for mapping.

Virus	Symptoms	ELISA	RT-PCR or PCR			Reference sequence accession no.
			Primers			
			Name	Sequence	Source	
**PEMV1**	Enation and mosaic	JKI-1841	HZ-355 r	5′-TCA GAA ATG ACG CCG GAA CA-3′	This study	NC_003629
			HZ-356 f	5′-GCG GAA CAA CCT GTC TCT GA-3’		
**PEMV2**			HZ-363 r	5′-GTT GTG CGT CCT CTT GGA GA-3′	This study	NC_003853
			HZ-366 f	5′-CCC AAG GAG GTG TCC ATG TC-3′		
**WHAV2**	NA	NA	HZ-576 r	5′-AGC GGA CAT CGA GAA AGC TT-3′	This study	NC_028382, NC_028383, NC_028386 and NC_028387
			HZ-577 f	5′-GAG AGC GTC AAT GGT GGT GA-3′		
**PNYDV**	Yellowing, dwarfing and leaf rolling	JKI-1604	priPeaSdir	5′-AAC CTC CGG ATA TCA CCA GAT-3′	[38,39]	NC_023154 to NC_023161
			priPeaSrev	5′-CCG GAG GTT TTA TTT CAA AAC CAA C-3′		
**VCV**	NA	NA	HZ-658 r	5′-GCG AGT TCA TCC GAA TGC AC-3′	This study	NC_007241 and NC_007242
			HZ-659 f	5′-GCC TCC AGT GAA GGT TTC GA-3′		
**PhCMoV**	Discoloration, mild yellow spots, distortion and fruit marbling	JKI-2051	HZ-343 r	5′-CGG TGA GTG GGG CAA CTA AT-3′	[20]	KY859866
			HZ-344 f	5′-AGC GAT GGG GTC TAG TGT CT-3′		
**PSTVd**	Symptomless	NA	primer A	5′-CCC TGA AGC GCT CCT CCG AG-3′	[40]	NC_002030
			primer B	5′-ATC CCC GGG GAA ACC TGG AGC GAA C-3′		

NA: Not applicable.

r: reverse.

f: forward.

### Nucleic acid extraction and virus/viroid enrichment

**Fig 1:**
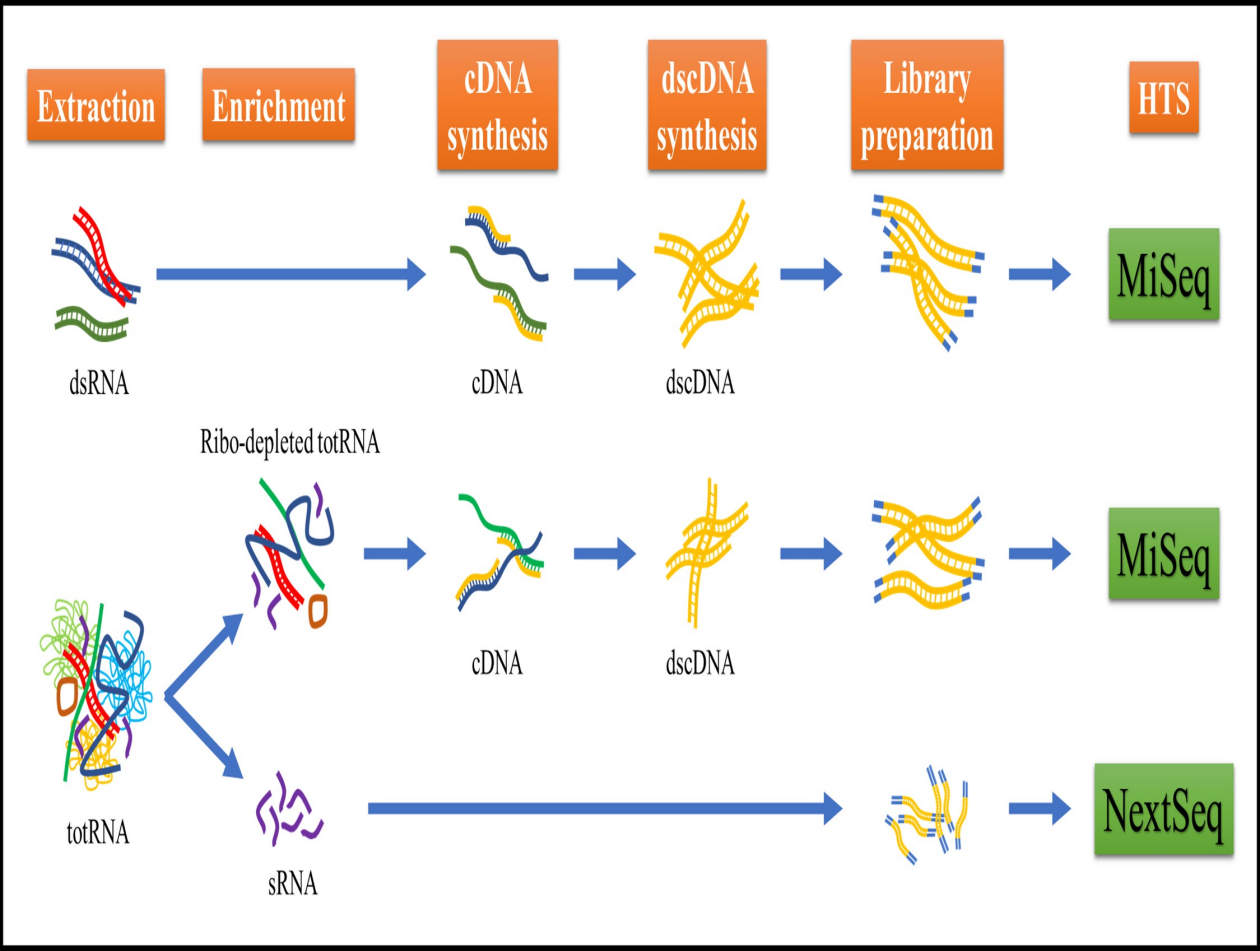
Graphical representation of the three RNA extractions (or enrichment) approaches used in this research, i.e. dsRNA, ribo-depleted totRNA and sRNA. The steps are indicated in orange boxes. The sequencing Illumina platforms are in green.

Five grams of leaf tissue from each infected plant were ground in liquid nitrogen and stored at -80°C until further extraction. For extraction, 100 mg leaf materials were mixed with 20 mg leaf discs from *P*. *vulgaris* infected with PvEV1 and PvEV2. The mix was used for three different RNA extraction methods (Fig 1):

- Double-stranded RNA extraction (dsRNA):
  dsRNA was extracted using Double-RNA Viral dsRNA Extraction Mini Kit (for plant tissue) (iNtRON Biotechnology, USA) according to the manufacturer’s protocol.
- Total RNA extraction followed by ribo-depletion (ribo-depleted totRNA):
  Total RNA extraction was performed using innuPREP Plant RNA Kit as described by the manufacturers’ instructions. The ribosomal RNA (rRNA) was depleted using the RiboMinus™ Plant Kit for RNA-Seq (Invitrogen) according to the manufacturers’ protocol.
- Total RNA extraction followed by small RNA extraction (sRNA):
  Total RNA was extracted as described above, then DNase treated using innuPREP DNase I Digest Kit (Analytik Jena AG) according to the manufacturers’ protocol. sRNA was extracted using polyacrylamide gel selection at Fasteris Life Sciences SA (Plan-les-Ouates, Switzerland).

Additionally, DNA extraction followed by rolling circle amplification (RCA) was carried out for the nanovirus infected plants. Genomic DNA was extracted as described before followed by RCA using TempliPhiTM 100 Amplification Kit (GE Healthcare Limited, UK).

### Nucleic acid preparations for HTS

For dsRNA and ribo-depleted totRNA, random cDNA was synthesized using ProtoScript II Reverse Transcriptase and random octamer primers (8N). A denaturation step of 99°C for 2 min for the dsRNA and 65°C for 5 min for the ribo-depleted totRNA. ds-cDNA was synthesized using NEBNext Ultra II Non-Directional RNA Second Strand Synthesis Module (New England Biolabs). Libraries were prepared using Nextera DNA Library Prep Kit (Illumina) following the manufacturer protocol. The quantification was done using Qubit dsDNA HS Assay Kit (Life Technologies) and quality analysis was done using High Sensitivity DNA Chips on Agilent 2100 Bioanalyzer (Agilent Technologies) following the manufacturers’ protocols. Subsequently, the libraries were sequenced on a MiSeq Illumina platform v.3 pair-end reads (2x301) at DSMZ (Braunschweig, Germany). For the sRNA, libraries were prepared from sRNA extracted using TruSeq small RNA kit (Illumina) at Fasteris Life Sciences SA (Plan-les-Ouates, Switzerland) and sequenced on a NextSeq Illumina platform single-end reads (1x50). For the RCA products, the library was also prepared using Nextera DNA Library Prep Kit and run on a NextSeq Illumina platform (2x151) at DSMZ (Braunschweig, Germany).

### Bioinformatic data analysis

The data analysis was performed using Geneious (version 11.1.5) (Biomatters Limited, Auckland, New Zealand). The adaptors and low-quality nucleotides were trimmed from the raw reads (quality score set to 0.05), then the trimmed reads were filtered by length (100 to 301nt for dsRNA and ribo-depleted totRNA; 20 to 24nt for sRNA). The filtered trimmed reads were *de novo* assembled using Geneious (parameters; Medium Sensitivity/Fast). Moreover, sRNA reads were also assembled using Velvet (*kmer* = 13/ minimum contig length = 30) [41].

The filtered-quality trimmed reads were also *kmer* normalised using BBNorm tool 37.64 (Brian Bushnell within Geneious) (parameters: Minimum depth = 5/ Target coverage level = 40 for MiSeq reads, and Minimum depth = 5/ Target coverage level = 100 for NextSeq reads). After that, the reads were *de novo* assembled as described above.

Assembled contigs were compared against a local database for viruses and viroids reference sequences using BLASTn (maximum E-value: 1e-5) downloaded 18 August 2018. To confirm the virus/viroid presence in each sample, the contigs were mapped to references (accession no. in Table 2). Additionally, filtered and trimmed reads were mapped to the reference sequences (Geneious; Medium Sensitivity/Fast and 5 iterations for dsRNA and ribo-depleted totRNA, and Medium-low Sensitivity/Fast and 5 iterations for sRNA). A cut-off for virus/viroid detection was set at ≥ 40% recovery of the reference sequence (for viruses) and ≥ 80% (for viroids). The consensus sequences were generated from the quality trimmed reads by mapping to reference sequences. The results were manually inspected to refine the ends of the genomes and consensus sequences were generated based on the quality of the nucleotides.

Pairwise nucleotide alignments were performed with ClustalW 2.1 (Cost matrix: CLUSTALW/ Gap open cost: 15/ Gap extend cost: 6.66) on Geneious while for protein alignments Clustal W 2.1 with parameters (Cost matrix: BLOSUM/ Gap open cost: 10/ Gap extend cost: 0.1).

### Comparing the three RNA-based approaches

The quality-controlled reads of each dataset were randomly subsampled (10 replicates) into the same number of reads (equal to approximately the same number of nucleotides 1, 10, 20, 30, 40 and 50 million nt). Resulting in a total of 720 subsets, each was used for *de novo* assembly and mapping to its reciprocal consensus sequence generated from the total reads. The number of nucleotides matched the references, percentage of the reference sequence recovered, and mean depth were calculated for each. Furthermore, *de novo* assembly (Geneious parameters; Medium Sensitivity/Fast) was performed for each subset and the resulting contigs were mapped to the corresponding consensus virus/viroid sequence and the percentages of whole genome was generated.

### Statistical analysis

The generated data from the bioinformatic analysis was statistically analysed using R version 3.5.1 [42]. The number of nucleotides matched the references, percentage of the reference sequence recovered by both reads and *de novo* assembled contigs, and mean depth were statistically compared. The data were visualised by ggplot2 and VennDiagram packages [43,44].

## Results

### Raw data

The statistics of the raw data generated from the HTS platforms of the three RNA-based approaches are indicated in S1 Table. As we used restricted quality and length of the reads, part of the generated datasets was of less quality than expected, this can be shown by the number of reads after trimming and filtering, and by the mean read length. Nevertheless, the datasets were still used for bioinformatic analysis. Furthermore, the internal controls (PvEV1 and PvEV2) that were used to spike the samples, were detected in all samples using the different approaches (S2 Table).

The cost of each approach was calculated on average based on our experience and the prices until early 2019 (S3 Table). The average cost of the dsRNA approach was about 307 Euros, the ribo-depleted totRNA costed 380 Euro, while the sRNA approach costed about 539 Euro on average.

### Virus/viroid detection

**Fig 2:**
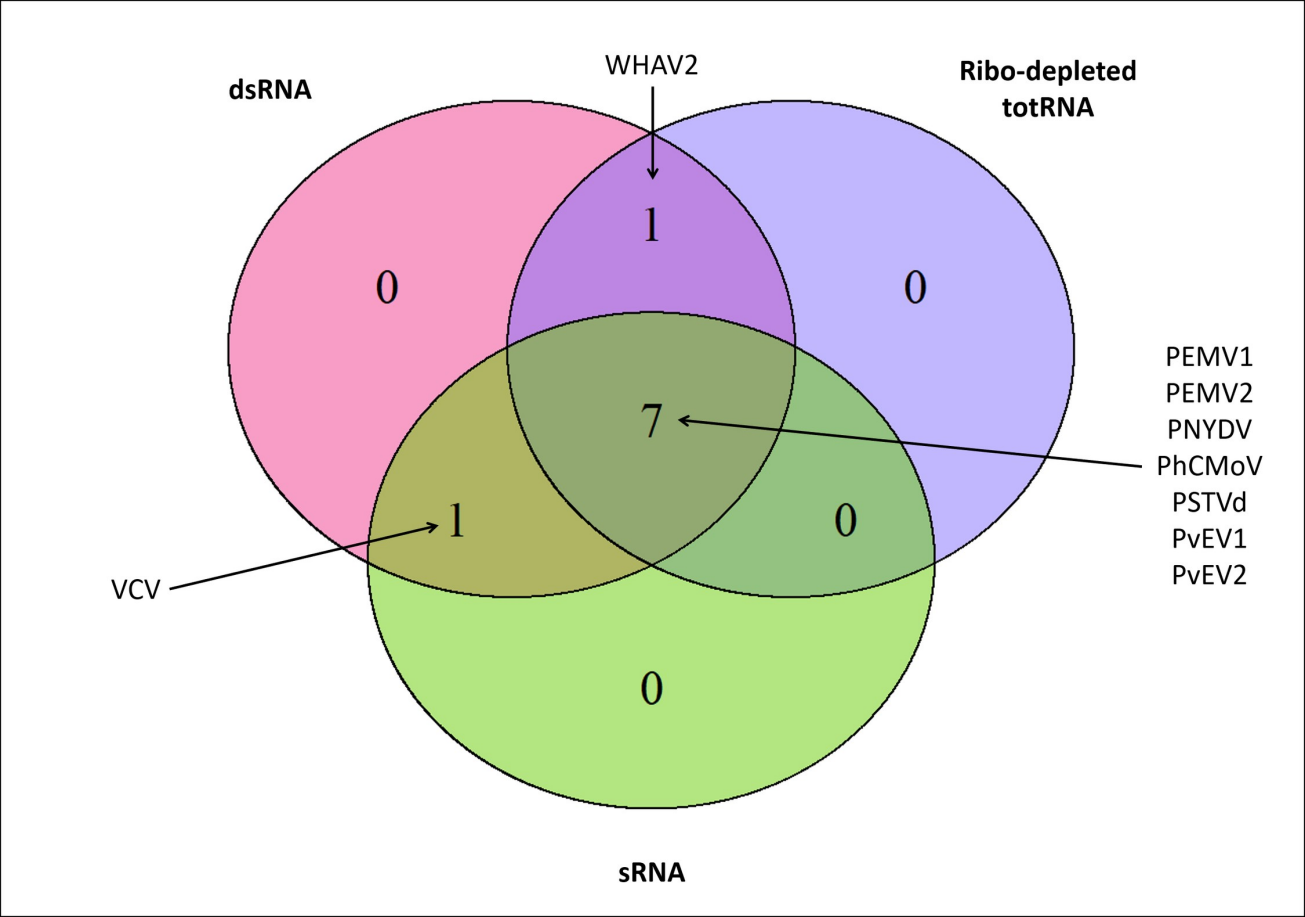
Venn diagram showing the viruses/viroid detected in all samples using different viral enrichment approaches (dsRNA, ribo-depleted totRNA and sRNA). The overlapping regions correspond to the number of viruses/viroid detected by more than one approach. The detected viruses were PEMV1: pea enation mosaic virus 1, PEMV2: pea enation mosaic virus 2, WHAV2: Wuhan aphid virus 2, PNYDV: pea necrotic yellow dwarf virus, VCV: Vicia cryptic virus and PhCMoV: Physostegia chlorotic mottle virus, PSTVd: potato spindle tuber viroid, PvEV1: Phaseolus vulgaris alphaendornavirus 1, and PvEV2: Phaseolus vulgaris alphaendornavirus 2.

Using BLASTn search, all three inoculated viruses and the viroid in this study were detected with all three RNA approaches, i.e. dsRNA, ribo-depleted totRNA and sRNA. Surprisingly, other viruses were also detected in sample 1 and sample 2. In sample 1, in addition to PEMV1, a divergent isolate of pea enation mosaic virus 2 (PEMV2) (Genus: *Umbravirus*/ Family: *Tombusviridae*) was detected by using all three approaches, and a divergent strain of Wuhan aphid virus 2 (WHAV2) was detected by dsRNA and ribo-depleted totRNA approaches (Fig 2). In sample 2, in addition to PNYDV, Vicia cryptic virus (VCV) (Genus: *Alphacryptovirus*/ Family: *Partitiviridae*) was also detected by dsRNA and sRNA approaches (Fig 2). These results showed that all viruses (expected and unexpected) were detected by the dsRNA approach while ribo-depleted RNA and sRNA approaches detected either WHAV2 or VCV, respectively. No associated RNAs or alphasatellites DNAs were identified in the different samples. The presence of PEMV2, WHAV2 and VCV were confirmed using RT-PCR and virus specific primers as listed in Table 2.

### Virus/viroid recovery

The total filtered and trimmed reads of each dataset were mapped to the different reference genomes of the nine viruses in the samples (six viruses, a viroid and the two spiked internal control viruses). The percentage of the total mapped reads and the percentage of the reference coverage can be found in Table 3. The numbers of mapped reads are different from one approach to another and from one virus to another. The full genomes of PEMV2, PhCMoV and PSTVd were recovered in the datasets by all three viral enrichment approaches. The almost complete genomes of PEMV1 (missing the 5' and 3' ends [not confirmed]/ length of the missing regions vary from method to another) were detected by the three approaches. The almost complete genomes of WHAV2 (missing the 5' and 3' ends of some segments [not confirmed]) were identified by both dsRNA and ribo-depleted totRNA approaches. For VCV, less than 90% of the genome was assembled by ribo-depleted totRNA and sRNA approaches, and for PNYDV, less than 80% of the genome was assembled by the three approaches (Table 3). Therefore, to obtain the full genome of PNYDV, RCA enrichment was used. All virus sequences were submitted to GenBank (accession no. MK948524 to MK948543).

**Table 3:** Viruses detected in each sample by the different approaches (dsRNA, ribo-depleted totRNA and sRNA) in the total quality filter and trimmed data.

Sample	Virus	dsRNA				Ribo-depleted totRNA				sRNA				Accession no.
		% of virus reads to total reads	Total no. of virus/viroid nt	% of ref.	Mean depth	reads	Total no. of virus/viroid nt	% of ref.	Mean depth	reads	Total no. of virus/viroid nt	% of ref.	Mean depth	
**1**	**PEMV1**	0.2	4,512,493	99.9	758.7	1.3	2,024,078	99.1	347.4	0.2	2,224,442	99.9	394.1	MK948533
	**PEMV2**	1.1	24,014,812	100	5,570.7	0.5	797,036	100	186.2	0.4	5,830,566	100	1,370.2	MK948534
	**WHAV2**	0.05	1,163,873	98.4	105.3	0.08	130,809	93.8	12	0	0	0	0	MK948535 to MK948538
**2**	**PNYDV**	0.004	39,512	77.2	4.9	0.01	38,587	72	4.6	0.02	215,596	63.5	27.1	MK948525 to MK948532
	**VCV**	0.01	51,391	89.4	13	0	0	0	0	0.01	67,266	87.5	17.4	MK948539 and MK948540
**3**	**PhCMoV**	0.8	9,737,744	100	725.3	15	37,885,237	100	3,750.4	0.5	8,081,770	100	606.8	MK948541
**4**	**PSTVd**	0.001	11,378	100	17.6	0.08	259,548	100	521.6	3.2	32,356,230	100	92,360.3	MK948524

The percentages (%) of the total number of mapped reads and of the recovered reference sequence, the total number of virus/viroid nucleotides (nt) and the mean depth are listed.

### Virus/viroid sequence characterisation

Pairwise nucleotide comparison between the sequences of PEMV1, PNYDV, PhCMoV and PSTVd showed that nucleotide (nt) identities range between 95.7% and 100% to their most similar sequence matches on NCBI. The French isolate of PEMV1 resulted in 95.7% identity with the ID isolate from Idaho USA (accession no. HM439775). The eight segments of PNYDV Elbtal isolate shared between 97.9% to 99.9% nt identities to their most similar sequences on NCBI. Segments DNA-N, -R, -S, -U4 shared 99.6% to 99.9% nt identities to the German isolate 110726 (accession no. KY810776 to KY810778 and KY810781). Segments DNA-C, -U1 and -U2 were most similar to the Danish isolate DK HZ16-582 by 97.9% to 99.4% (accession no. MH000257, MH000258 and MH000260), and segment DNA-M is most similar to the Danish isolate DK HZ16-573 by 98.6% (accession no. MH000250). PhCMoV HZ16-558 isolate shared 99.6% nt identity to HZ15-192 isolate (accession no. KY859866). The PSTVd isolate shared 100% nt identity with isolates 6718566 from Netherlands and 07087900 from Belgium (accession no. KX370618 and FM998548, respectively). The sequences of PEMV2 and WHAV2 isolates were divergent from the reference sequences (S4 and S5 Tables). The predicted proteins of PEMV2 shared between 92.5% to 97.6% aa identities to their analogues of the most similar isolate from the UK (S4 Table) whereas WHAV2 protein sequences shared 90.2% to 96.8% aa identities to the predicted proteins of the Chinese strain WHYC-2 (S5 Table). PvEV1 shared 99.7% nt identity to the Mexican isolate INIFAP CG1 (accession no. MG640415) and PvEV2 shared highest nt identity with a Brazilian isolate with 99.4% (accession no. AB719398).

### The sensitivity of the three approaches (virus nucleotides and average depth)

**Fig 3:**
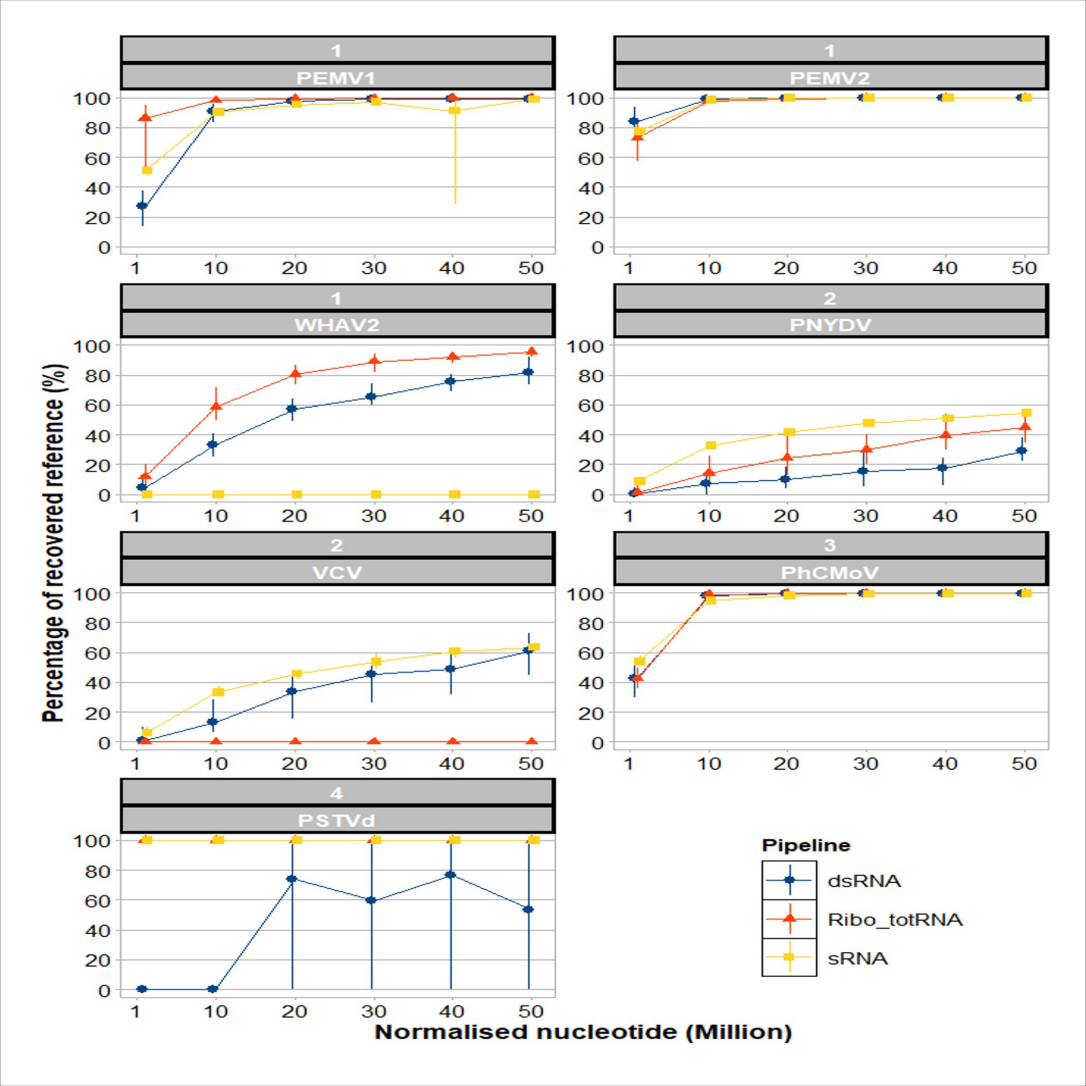
Percentage of reference sequences recovered by the reads of the RNA-based approaches on each of the normalised nucleotide subsamples (sizes: 1M, 10M, 20M, 30M, 40M and 50M nt). The means of each approach are shown as blue circle: dsRNA, red triangle: ribo-depleted totRNA and yellow square: sRNA. The means are joined by lines with same colours. The vertical lines represent the standard deviation of the ten replicates. The strips over each graph are divided into two parts (upper: sample number, lower: virus/viroid acronym).

The sensitivity of each approach for the detection of the viruses/viroid in this study was analysed using normalised subsamples (sizes: 1M, 10M, 20M, 30M, 40M and 50M nt). Each of the normalised subsamples from the different sizes was mapped to the consensus sequences of the detected viruses/viroid. The percentages of recovered references (here the consensus sequences) by the viral/viroid nt of each subsample are shown in Fig 3. With the dsRNA approach, the recovered percentages of the genomes reached 100% at 10M nt for PEMV2 and PhCMoV, whereas PEMV1 was only recovered at 20M nt. In the case of PSTVd, the full genome was recovered in some replicates from 20M to 50M nt, but the means were lower than 80%. However, for the segmented viruses: WHAV2, PNYDV and VCV, the whole genomes could not be recovered even when using 50M nt. Using ribo-depleted totRNA, the full genomes of PEMV1, PEMV2 and PhCMoV recovered at 10M nt (Fig 3). The full genome of PSTVd was recovered at 1M nt. Similar to dsRNA approach, the genomes of WHAV2 and PNYDV were not recovered in all the subsamples. sRNA performed similar to dsRNA approach with the viruses they both detected. PSTVd recovered at 1M nt, PEMV2 and PhCMoV at 10M, PEMV1 at 20M, and PNYDV reached about 60% of the genomes at 50M nt. Additionally, VCV also reached about 60% of the genomes at 50M nt.

**Fig 4:**
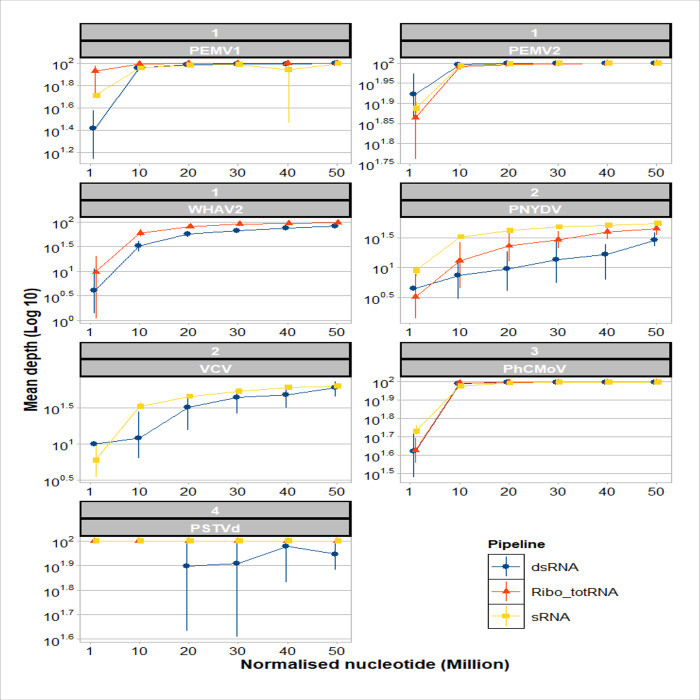
Mean depth of the RNA-based approaches for the detection of the viruses/viroid in this study on each of the normalised nucleotide depth (1M, 10M, 20M, 30M, 40M and 50M nt) for each subsample. The means of each approach are shown as blue circle: dsRNA, red triangle: ribo-depleted totRNA and yellow square: sRNA. The means are joined by lines with same colours. The vertical lines represent the standard deviation of the ten replicates. The strips over each graph are divided into two parts (upper: sample number, lower: virus/viroid acronym).

The dsRNA normalised subsamples had low variation for PEMV1, PEMV2, WHAV2 and PhCMoV, slight variation for PNYDV and VCV, and high variation in case of PSTVd (Fig 3). For ribo-depleted totRNA, all the replicates in all the viruses had low variation except for PNYDV showed slight variation. sRNA had low variation except in one subsample of PEMV1, i.e. size 40M nt. The generated contigs by *de novo* assembly of each normalised subsamples showed the same results (S1 Fig). In Fig 4, the mean depth increased with the size of subsamples in all the three RNA approaches. Regarding the variation, it was the same as the percentages of recovered references of Fig 3.

## Discussion

The three viral enrichment approaches (dsRNA, ribo-depleted totRNA and sRNA) used here enabled the detection and identification of the known and unknown plant viruses/viroid in this study. The suitability of the three approaches for virus detection by HTS was confirmed by the recovery of positive internal controls (PvEV1 and PvEV2) that were used for spiking the samples. Using the MiSeq platform in two directions (forward and reverse), there were low-quality reads, mainly based on the low quality of the reverse sequencing reads. These low-quality reads did not affect the analyses as we recovered sufficient reads and we used normalised reads for the analyses. In addition, we recovered our internal control sequences used to spike the extracts. A different sequencing platform, e.g. HiSeq, would have resulted in higher sequencing quality for the reverse strand but would have produced shorter reads.

The dsRNA approach was more efficient than the ribo-depleted totRNA and the sRNA approaches in terms of virus detection. This is because all the eight viruses and the one viroid in this study were detected by the dsRNA approach, while each of the ribo-depleted totRNA and the sRNA approaches, only detected seven viruses and the viroid. Three of the detected viruses were not known to be in the samples, i.e. PEMV2, VCV and WHAV2.

The detection of PEMV2 in mixed infection with PEMV1 is well documented [45]. Both viral genomes were recovered with high sequencing depth in all three approaches. VCV is a cryptic (symptomless) virus which occurs at very low concentrations in infected tissues of several German varieties of *V*. *faba* [46]. VCV was detected by the dsRNA and sRNA approaches. Furthermore, the ability of HTS to detect cryptic viruses was reported before in [47]. Interestingly, four segments of a divergent strain of WHAV2 were identified in sample 1 by dsRNA and ribo-depleted totRNA. WHAV2 was discovered in *Hyalopterus pruni* and *Aulacorthum magnoliae* aphids from Wuhan, Hubei province, China in 2013 [48,49]. It has a segmented linear (+ve) ssRNA genome, its virion structure is unknown, and it is not assigned to a virus family yet. However, WHAV2 was phylogenetically grouped in the Jingmenviruses clade [49]. Moreover, WHAV2 segments were also detected in three other pea samples collected from Germany and Austria in 2012 and 2013 (data not published). This considered the first detection of WHAV2 sequences in a plant host and is also the first record of this finding in Europe.

Concerning the recovered number of viral/viroid nt and the mean depth, in general, their amounts were different depending on the species, the sample and the approach. The recovered viral sequences from the complete data were comparatively low for all samples which might be explained by early sampling and therefore low virus titre. As expected, the number of viral/viroid nt and the mean depth increased with the increase in the size of subsamples. Similarly, the size of the generated contigs and their genome coverage increased with the size of the subsamples.

Linear monopartite ssRNA genomes, i.e. PEMV1, PEMV2 and PhCMoV, were detected using all approaches. When the viral nt were mapped to the single-stranded linear non-segmented consensus sequences (positive or negative), 10M to 20M nt were enough to recover the complete viral genomes. As dsRNAs are produced during viral replication, extraction of dsRNA may enhance the complete recovery of these genomes. Similarly, the removal of host ribosomal RNAs increases the viral/viroid reads compared to the host sequence data. As RNA silencing is a defence mechanism of plants against viral infections, a high amount of viral sRNA is produced upon infection and can be recovered by small RNA approaches.

For the viroid PSTVd, as it has a short nt sequence (360nt), 1M nt were enough to recover the full genome when ribo-depleted totRNAs and sRNAs were used, but this did not work for the dsRNAs where more than 20M nt were required here. It might be possible that PSTVd accumulates to high titres in infected tissues thus leading to an increased concentration of ss- and ds- viroid RNAs. Both RNA forms can serve as templates for the Dicer of the RNA silencing complex thus leading to degradation of these molecules and the production of sRNAs [50]. These may explain the very high PSTVd nt recovered by the sRNA approach in comparison to dsRNA and ribo-depleted totRNA.

Segmented viruses, regardless of the genomic composition (nucleic acid type, size and number of the segments), were not well recovered by the three approaches at a normalised nucleotide depth lower than 50M nt. VCV was only detected by using the dsRNA and sRNA approaches. The number of viral nt in the two approaches was not significantly different. The low concentration of cryptic viruses such as VCV and its dsRNA genome could be the main reasons why it was not recovered using ribo-depleted totRNA. In the case of dsRNA approach, the dsRNAs are denatured at 99°C prior to reverse transcription to allow primer binding. However, in the case of ribo-depleted totRNA approach a denaturation step was only carried at 65°C which may not be sufficient to denature the dsRNA molecules of VCV. As WHAV2 might be an aphid virus that is using plants as a vector, this may explain its low titre in planta [49].

The DNA virus in this study, PNYDV, was not recovered completely using the three RNA approaches although a slightly higher number of nt could be recovered in case of the sRNA approach. As PNYDV has a circular ssDNA multipartite genome, mRNA must be synthesised for protein translation. Nanoviruses are not known to produce dsRNA. However, in previous research DNA viruses were discovered using dsRNA extraction approaches albeit as low sequence reads [23]. As the virus is a phloem restricted virus, a low titre is expected [51]. These might be the reasons for the lower recovery of the virus nt which has also been observed by Pecman and colleagues [34].

Taking sample preparation into consideration, dsRNA extraction consumed less time (<1 hour) in comparison to total RNA extraction followed by ribodepletion (about 4 hours) and total RNA followed by sRNA extraction (about 6 hours). The libraries were then sequenced on Illumina platforms MiSeq for both dsRNA and ribo-depleted totRNA, while sRNA on a NextSeq platform but turnaround time was dependent on the sequencing provider.

The costs of dsRNA extraction per sample was less than for the other two approaches (S3 Table). The costs were calculated based on our experience and the prices at the time. Increasing the number of samples per lane will reduce the costs of the sequencing platform runs, this was also concluded by [34]. Moreover, multiplexing by the use of additional barcodes before library preparation allows the sequencing of more samples per run and consequently will reduce the costs to a more comparable price [23]. However, in our own experience, the use of barcodes can be challenging due to barcode hopping and/or difficulties in barcode splitting.

Our results suggest that for successful plant virus/viroid detection using HTS, sequencing of dsRNA might be the most suitable approach when a sequencing depth of at least a 130,000 of high-quality reads are chosen. The ability of dsRNA enrichment to detect ssRNA (positive and negative), dsRNA and DNA viruses, and viroids was reported before [10,52]. Since most plant RNA viruses produce a dsRNA molecule as replication intermediate and some viruses have a dsRNA genome, and that dsRNA is very stable and can be easily purified, sequencing of dsRNA is therefore a very powerful method for detecting all virus types. If enough sequencing depth is applied, DNA viruses can also be recovered using this dsRNA extraction approach, most likely due to the co-purification of DNA-RNA hybrid molecules. There are different dsRNA extraction methods available for HTS. We used the dsRNA extraction kit as it consumes less time (<1 hour), uses less milligrams (50 to 200) of plant tissue as starting material, nor does it require a PCR amplification step that would be required for other dsRNA extraction methods [53–55]. In addition, it is very cost-effective per sample.

The study further highlights the ability of HTS to detect known and unknown plant viruses and viroids. This study showed that the performance of the three RNA-based approaches is virus/viroid and sample dependent. We conclude that HTS generated data from the dsRNA approach outcompeted the ones generated from ribo-depleted totRNA and sRNA, and potentially can be used for the detection of all plant viruses and viroids if sufficient sequence data is used.

## Supporting information

S1 Fig Percentage of reference sequences recovered by the produced contigs of the RNA-based approaches on each of the normalised nucleotide subsamples (sizes: 1M, 10M, 20M, 30M, 40M and 50M nt). The means of each approach are shown as blue circle: dsRNA, red triangle: ribo-depleted totRNA and yellow square: sRNA. The means are joined by lines with same colours. The vertical lines represent the standard deviation of the ten replicates. The strips over each graph are divided into two parts (upper: sample number, lower: virus/viroid acronym).
(TIF)

S1 Table Raw data statistics of the generated reads by using the three RNA approaches (dsRNA, ribo-depleted totRNA and sRNA). (DOCX)

S2 Table Analysis of the spiked cryptic viruses Phaseolus vulgaris alphaendornavirus 1 and 2 (PvEV1 and PvEV2) in each library of the four samples with the three RNA approaches. (DOCX)

S3 Table Average costs per sample of the three approaches used in the study. Costs presented in Euros.
(DOCX)

S4 Table Pairwise comparisons of the nucleotide (nt) sequences and the amino acid (aa) sequence identities of Fr HZ11-065 isolates of PEMV1 and PEMV2 proteins with their most similar known isolates. (DOCX)

S5 Table Pairwise comparisons of the four segments of the French isolate of WHAV2 (Fr HZ11-065) based on the nucleotide (nt) sequences and the amino acid (aa) sequences of their predicted proteins. (DOCX)

## References

[1] Anderson PK, Cunningham AA, Patel NG, Morales FJ, Epstein PR et al (2004) Emerging infectious diseases of plants: Pathogen pollution, climate change and agrotechnology drivers. Trends Ecol Evol 19: 535–544. 10.1016/j.tree.2004.07.021 16701319

[2] Canto T, Aranda MA, Fereres A (2009) Climate change effects on physiology and population processes of hosts and vectors that influence the spread of hemipteran-borne plant viruses. Glob Change Biol 15: 1884–1894.

[3] Hulme PE (2017) Climate change and biological invasions: evidence, expectations, and response options. Biol Rev 92 (3): 1297–1313. 10.1111/brv.12282 27241717

[4] Jones RAC (2016) Future scenarios for plant virus pathogens as climate change progresses. Adv Vir Res 95: 87–147. 10.1016/bs.aivir.2016.02.004 27112281

[5] Myers SS, Smith MR, Guth S, Golden CD, Vaitla B et al (2017) Climate change and global food systems: potential impacts on food security and undernutrition. Ann Rev Public Health 38: 259–277. 28125383 10.1146/annurev-publhealth-031816-044356

[6] Ward E, Foster SJ, Fraaije BA, Mccartney HA (2004) Plant pathogen diagnostics: immunological and nucleic acid-based approaches. Ann Appl Biol 145 (1): 1–16.

[7] Richert-Pöggeler KR, Franzke K, Hipp K, Kleespies RG (2018) Electron microscopy methods for virus diagnosis and high resolution analysis of viruses. Front Microbiol 9: 3255 10.3389/fmicb.2018.03255 30666247 PMC6330349

[8] Adams I, Fox A (2016) Diagnosis of plant viruses using next-generation sequencing and metagenomic analysis In: Wang A, Zhou X, editors. Current research topics in plant virology. Cham, Switzerland: Springer pp. 323–335.

[9] Gaafar Y, Sieg-Müller A, Lüddecke P, Hartrick J, Seide Y et al (2019) First report of natural infection of beetroot with Beet soil-borne virus. New Dis Rep 40: 5.

[10] Rott M, Xiang Y, Boyes I, Belton M, Saeed H et al (2017) Application of next generation sequencing for diagnostic testing of tree fruit viruses and viroids. Plant Dis 101 (8): 1489–1499. 10.1094/PDIS-03-17-0306-RE 30678581

[11] Adams IP, Miano DW, Kinyua ZM, Wangai A, Kimani E et al (2013) Use of next-generation sequencing for the identification and characterization of Maize chlorotic mottle virus and Sugarcane mosaic virus causing maize lethal necrosis in Kenya. Plant Pathol 62 (4): 741–749.

[12] Roossinck MJ (2017) Deep sequencing for discovery and evolutionary analysis of plant viruses. Virus Res 239: 82–86. 10.1016/j.virusres.2016.11.019 27876625

[13] Villamor DEV, Ho T, Al Rwahnih M, Martin RR, Tzanetakis IE (2019) High throughput sequencing for plant virus detection and discovery. Phytopathology 109 (5): 716–725. 10.1094/PHYTO-07-18-0257-RVW 30801236

[14] Elbeaino T, Digiaro M, Uppala M, Sudini H (2015) Deep sequencing of dsRNAs recovered from mosaic-diseased pigeonpea reveals the presence of a novel emaravirus: pigeonpea sterility mosaic virus 2. Arch Virol 160 (8): 2019–2029. 10.1007/s00705-015-2479-y 26060057

[15] Chen S, Jiang G, Wu J, Liu Y, Qian Y et al (2016) Characterization of a novel polerovirus infecting maize in China. Viruses 8 (5). 10.3390/v8050120 PMC4885075 27136578

[16] Villamor DEV, Pillai SS, Eastwell KC (2017) High throughput sequencing reveals a novel fabavirus infecting sweet cherry. Arch Virol 162 (3): 811–816. 10.1007/s00705-016-3141-z 27815695

[17] Gaafar YZA, Richert-Pöggeler KR, Sieg-Müller A, Lüddecke P, Herz K et al (2019) Caraway yellows virus, a novel nepovirus from Carum carvi. Virol J 16 (1): 529. 10.1186/s12985-019-1181-1 PMC6537451 31133023

[18] Maree HJ, Fox A, Al Rwahnih M, Boonham N, Candresse T (2018) Application of HTS for routine plant virus diagnostics: state of the art and challenges. Front Plant Sci 9: 1082 10.3389/fpls.2018.01082 30210506 PMC6119710

[19] Leff JW, Lynch RC, Kane NC, Fierer N (2017) Plant domestication and the assembly of bacterial and fungal communities associated with strains of the common sunflower, *Helianthus annuus*. New Phytol 214 (1): 412–423. 10.1111/nph.14323 27879004

[20] Gaafar YZA, Abdelgalil MAM, Knierim D, Richert-Pöggeler KR, Menzel W et al (2018) First report of physostegia chlorotic mottle virus on tomato (Solanum lycopersicum) in Germany. Plant Dis 102 (1): 255.

[21] Hull R (2009) Chapter 6—Plant viral genomes In: Hull R, editor. Comparative Plant Virology. Amsterdam, Boston: Elsevier/Academic Press.

[22] Morris TJ (1979) Isolation and analysis of double-stranded RNA from virus-infected plant and fungal tissue. Phytopathology 69 (8): 854. Accessed 3 January 2019. 10.1094/Phyto-69-854 40934407

[23] Roossinck MJ, Saha P, Wiley GB, Quan J, White JD et al (2010) Ecogenomics: using massively parallel pyrosequencing to understand virus ecology. Mol Ecol 19 Suppl 1: 81–88. 20331772 10.1111/j.1365-294X.2009.04470.x

[24] Bar-Joseph M, Rosner A, Moscovitz M, Hull R (1983) A simple procedure for the extraction of double-stranded RNA from virus-infected plants. J Virol Methods 6 (1): 1–8. 10.1016/0166-0934(83)90062-9 6833446

[25] Gaafar YZA, Sieg-Müller A, Lüddecke P, Herz K, Hartrick J et al (2019) First report of Turnip crinkle virus infecting garlic mustard (Alliaria petiolata) in Germany. New Dis Rep 39: 9.

[26] Kreuze JF, Perez A, Untiveros M, Quispe D, Fuentes S et al (2009) Complete viral genome sequence and discovery of novel viruses by deep sequencing of small RNAs: a generic method for diagnosis, discovery and sequencing of viruses. Virology 388 (1): 1–7. 10.1016/j.virol.2009.03.024 19394993

[27] Gaafar YZA, Richert-Pöggeler KR, Maaß C, Vetten H-J, Ziebell H (2019) Characterisation of a novel nucleorhabdovirus infecting alfalfa (Medicago sativa). Virol J 16 (1): 55 10.1186/s12985-019-1147-3 31036009 PMC6489223

[28] Wyant PS, Strohmeier S, Schäfer B, Krenz B, Assunção IP et al (2012) Circular DNA genomics (circomics) exemplified for geminiviruses in bean crops and weeds of northeastern Brazil. Virology 427 (2): 151–157. 10.1016/j.virol.2012.02.007 22397740

[29] Gaafar YZA, Nielsen GC, Ziebell H (2018) Molecular characterisation of the first occurrence of Pea necrotic yellow dwarf virus in Denmark. New Dis Rep 37: 16.

[30] Flores R, Di Serio F, Hernández C (1997) Viroids: The noncoding Genomes. Seminars in Virology 8 (1): 65–73.

[31] Steger G, Riesner D (2003) Molecular characteristics In: Hadidi A, Flores R, Randles J, Semancik J, editors. Viroids: Properties, Detection, Diseases and their Control: CSIRO Publishing pp. 15–29.

[32] Flores R, Gas M-E, Molina-Serrano D, Nohales M-Á, Carbonell A et al (2009) Viroid replication: Rolling-circles, enzymes and ribozymes. Viruses 1 (2): 317–334. 10.3390/v1020317 21994552 PMC3185496

[33] Visser M, Bester R, Burger JT, Maree HJ (2016) Next-generation sequencing for virus detection: covering all the bases. Virol J 13: 85 10.1186/s12985-016-0539-x 27250973 PMC4890495

[34] Pecman A, Kutnjak D, Gutiérrez-Aguirre I, Adams I, Fox A et al (2017) Next generation sequencing for detection and discovery of plant viruses and viroids: comparison of two approaches. Front Microbiol 8: 1998 10.3389/fmicb.2017.01998 29081770 PMC5645528

[35] Clark MF, Adams AN (1977) Characteristics of the microplate method of enzyme-linked immunosorbent assay for the detection of plant viruses. J Gen Virol 34 (3): 475–483. 10.1099/0022-1317-34-3-475 323416

[36] Fletcher J, Tang J, Blouin A, Ward L, MacDiarmid R et al (2016) Red clover vein mosaic virus—a novel virus to New Zealand that is widespread in legumes. Plant Dis 100 (5): 890–895. 10.1094/PDIS-04-15-0465-RE 30686157

[37] Edwards K, Johnstone C, Thompson C (1991) A simple and rapid method for the preparation of plant genomic DNA for PCR analysis. Nucleic Acids Res 19 (6): 1349 10.1093/nar/19.6.1349 2030957 PMC333874

[38] Gaafar YZA, Grausgruber-Gröger S, Ziebell H (2016) *Vicia faba*, *V*. *sativa* and *Lens culinaris* as new hosts for *Pea necrotic yellow dwarf virus* in Germany and Austria. New Dis Rep 34: 28.

[39] Gaafar YZA, Timchenko T, Ziebell H (2017) First report of *Pea necrotic yellow dwarf virus* in The Netherlands. New Dis Rep 35: 23.

[40] Weidemann H-L, Buchta U (1998) A simple and rapid method for the detection of potato spindle tuber viroid (PSTVd) by RT-PCR. Potato Res 41 (1): 1–8.

[41] Zerbino DR, Birney E (2008) Velvet: algorithms for de novo short read assembly using de Bruijn graphs. Genome Res 18 (5): 821–829. 10.1101/gr.074492.107 18349386 PMC2336801

[42] R Core Team (2014) R: a language and environment for statistical computing.

[43] Chen H, Boutros PC (2011) VennDiagram: A package for the generation of highly-customizable Venn and Euler diagrams in R. BMC Bioinformatics 12: 35 10.1186/1471-2105-12-35 21269502 PMC3041657

[44] Ginestet C (2011) ggplot2: elegant graphics for data analysis. J R Stat Soc Series B Stat Methodol 174 (1): 245–246.

[45] Doumayrou J, Sheber M, Bonning BC, Miller WA (2017) Quantification of *Pea enation mosaic virus* 1 and 2 during infection of *Pisum sativum* by one step real-time RT-PCR. J Virol Methods 240: 63–68. 10.1016/j.jviromet.2016.11.013 27915037

[46] Blawid R, Stephan D, Maiss E (2007) Molecular characterization and detection of Vicia cryptic virus in different *Vicia faba* cultivars. Arch Virol 152 (8): 1477–1488. 10.1007/s00705-007-0966-5 17533556

[47] Roossinck MJ (2011) The good viruses: viral mutualistic symbioses. Nat Rev Microbiol 9 (2): 99–108. 10.1038/nrmicro2491 21200397

[48] Li C-X, Shi M, Tian J-H, Lin X-D, Kang Y-J et al (2015) Unprecedented genomic diversity of RNA viruses in arthropods reveals the ancestry of negative-sense RNA viruses. eLife 4. 10.7554/eLife.05378 PMC4384744 25633976

[49] Shi M, Lin X-D, Vasilakis N, Tian J-H, Li C-X et al (2016) Divergent viruses discovered in arthropods and vertebrates revise the evolutionary history of the Flaviviridae and related viruses. J Virol 90 (2): 659–669. 10.1128/JVI.02036-15 26491167 PMC4702705

[50] Markarian N, Li HW, Ding SW, Semancik JS (2004) RNA silencing as related to viroid induced symptom expression. Arch Virol 149 (2): 397–406. 10.1007/s00705-003-0215-5 14745603

[51] Vetten HJ, Dale JL, Grigoras I, Gronenborn B, Harding R et al. (2011) Family Nanoviridae. In: King AMQ, Lefkowitz E, Adams MJ, Carstens EB, editors. Virus taxonomy. Ninth report of the International Committee on Taxonomy of Viruses / edited by Andrew King. Oxford: Elsevier. pp. 395–404.

[52] Rott ME, Kesanakurti P, Berwarth C, Rast H, Boyes I et al (2018) Discovery of negative-sense RNA viruses in trees infected with apple rubbery wood disease by next-generation sequencing. Plant Dis 102 (7): 1254–1263. 10.1094/PDIS-06-17-0851-RE 30673558

[53] Kesanakurti P, Belton M, Saeed H, Rast H, Boyes I et al (2016) Screening for plant viruses by next generation sequencing using a modified double strand RNA extraction protocol with an internal amplification control. J Virol Methods 236: 35–40. 10.1016/j.jviromet.2016.07.001 27387642

[54] Yanagisawa H, Tomita R, Katsu K, Uehara T, Atsumi G et al (2016) Combined DECS analysis and next-generation sequencing enable efficient detection of novel plant RNA viruses. Viruses 8 (3): 70 10.3390/v8030070 27072419 PMC4810260

[55] Blouin AG, Ross HA, Hobson-Peters J, O'Brien CA, Warren B et al (2016) A new virus discovered by immunocapture of double-stranded RNA, a rapid method for virus enrichment in metagenomic studies. Mol Ecol Resour 16 (5): 1255–1263. 10.1111/1755-0998.12525 26990372

