# Supplementary figures and images for: Comparative study on three viral enrichment approaches based on RNA extraction for plant virus/viroid detection using high-throughput sequencing
Source: PLoS One. 2020 Aug 25;15(8):e0237951. doi: 10.1371/journal.pone.0237951 (PMC7447037; doi:10.1371/journal.pone.0237951)

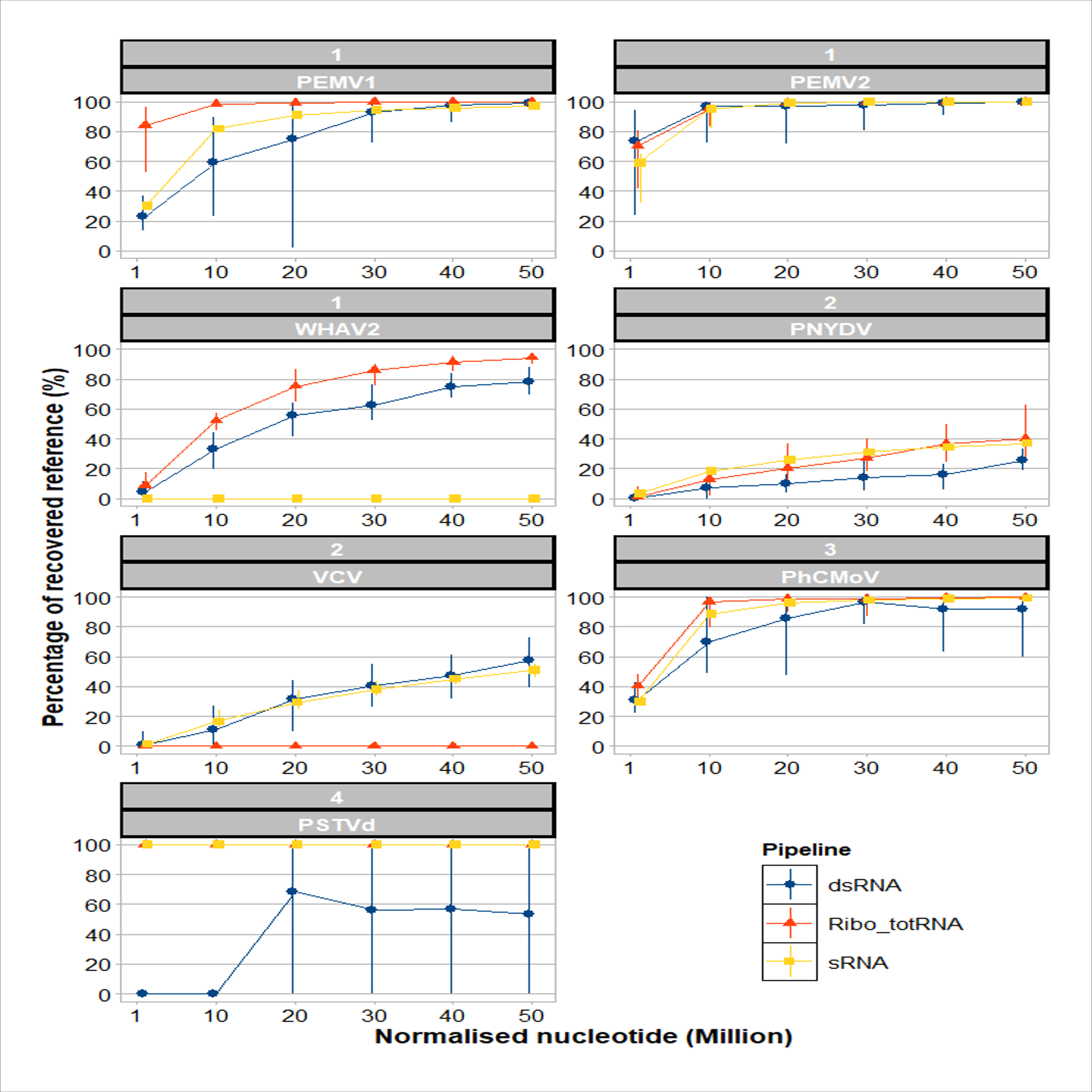

Supplement: S1 Fig — The means of each approach are shown as blue circle: dsRNA, red triangle: ribo-depleted totRNA and yellow square: sRNA. The means are joined by lines with same colours. The vertical lines represent the standard deviation of the ten replicates. The strips over each graph are divided into two parts (upper: sample number, lower: virus/viroid acronym). (TIF) [file pone.0237951.s001.tif]
