# Supplementary material for: Comparative study on three viral enrichment approaches based on RNA extraction for plant virus/viroid detection using high-throughput sequencing
Source: PLoS One. 2020 Aug 25;15(8):e0237951. doi: 10.1371/journal.pone.0237951 (PMC7447037; doi:10.1371/journal.pone.0237951)
Supplement: S1 Table — (DOCX) [file pone.0237951.s002.docx]

**S1 Table. Raw data statistics of the generated reads by using the three RNA approaches (dsRNA, ribo-depleted totRNA and sRNA).**

| **Approach** | | **dsRNA** | | | | **Ribo-depleted totRNA** | | | | **sRNA** | | | |
| --- | --- | --- | --- | --- | --- | --- | --- | --- | --- | --- | --- | --- | --- |
| **Sample** | | **1** | **2** | **3** | **4** | **1** | **2** | **3** | **4** | **1** | **2** | **3** | **4** |
| **Total raw reads** | | 9,950,706 | 3,544,494 | 5,569,228 | 3,393,744 | 835,294 | 1,721,612 | 1,402,288 | 1,723,250 | 66,643,808 | 41,625,899 | 69,053,975 | 47,036,623 |
| **Raw data quality** | **≥Q20** | 90.1% | 84.5% | 91.6% | 91.5% | 96.20% | 96.50% | 96.10% | 95.30% | 94.60% | 94.60% | 94.80% | 95.20% |
|  | **≥Q30** | 80.3% | 71.7% | 82.7% | 81.9% | 89.40% | 89.90% | 89.20% | 87.20% | 92.60% | 92.80% | 92.80% | 93.50% |
| **Quality and size filtered/trimmed reads** | | 5,987,048 | 1,692,308 | 3,330,634 | 2,226,748 | 471,574 | 1,039,438 | 826,984 | 1,037,554 | 11,370,176 | 9,565,289 | 19,291,514 | 21,828,752 |
| **Read length**  **Mean ± Std. Dev.**  **(quality trimmed)** | | 228.4 ± 65.0 | 258.7 ± 56.7 | 238.7 ± 63.5 | 255.0 ± 58.4 | 176.7 ± 49.7 | 180.1 ± 50.6 | 179.6 ± 50.2 | 187.7 ± 51.6 | 21.8 ± 1.3 | 22.0 ± 1.4 | 22.0 ± 1.4 | 21.7 ± 1.3 |
