# Supplementary material for: Comparative study on three viral enrichment approaches based on RNA extraction for plant virus/viroid detection using high-throughput sequencing
Source: PLoS One. 2020 Aug 25;15(8):e0237951. doi: 10.1371/journal.pone.0237951 (PMC7447037; doi:10.1371/journal.pone.0237951)
Supplement: S2 Table — (DOCX) [file pone.0237951.s003.docx]

**S2 Table. Analysis of the spiked cryptic viruses Phaseolus vulgaris alphaendornavirus 1 and 2 (PvEV1 and PvEV2) in each library of the four samples with the three RNA approaches**

| **Virus** | **Sample** | **dsRNA** | | | | **Ribo-depleted totRNA** | | | | **sRNA** | | | | **Reference** |
| --- | --- | --- | --- | --- | --- | --- | --- | --- | --- | --- | --- | --- | --- | --- |
|  |  | **No. of nt** | **no of reads** | **% of ref.** | **Mean depth** | **No. of nt** | **no of reads** | **% of ref.** | **Mean depth** | **No. of nt** | **no of reads** | **% of ref.** | **Mean depth** |  |
| **PvEV1** | **1** | 5,082,751 | 22,050 | 99.1 | 356 | 1,745 | 10 | 6.6 | 0.1 | 24,455 | 1,122 | 43.3 | 1.7 | NC_039217 |
|  | **2** | 968,340 | 3,715 | 98.9 | 67.9 | 4,434 | 23 | 15.7 | 0.3 | 2,335 | 107 | 10.8 | 0.2 |  |
|  | **3** | 5,208,700 | 21,435 | 99.3 | 366.8 | 706 | 4 | 2.5 | <0.1 | 72,613 | 3,345 | 41.6 | 5.16 |  |
|  | **4** | 2,724,640 | 18,048 | 99.5 | 322.9 | 645 | 4 | 2.2 | <0.1 | 28,008 | 1,291 | 33.2 | 2 |  |
| **PvEV2** | **1** | 2,881,894 | 12,654 | 99.9 | 190.5 | 596 | 4 | 2 | <0.1 | 52,719 | 2,446 | 48.7 | 3.6 | NC_038422 |
|  | **2** | 451,820 | 1,733 | 99.2 | 29.5 | 1,902 | 10 | 5.3 | 0.1 | 4,264 | 197 | 16.6 | 0.3 |  |
|  | **3** | 2,925,625 | 12,124 | 100 | 193.1 | 2,611 | 6 | 4.4 | 0.1 | 188,153 | 8,749 | 55.4 | 12.7 |  |
|  | **4** | 4,689,607 | 10,491 | 99.7 | 179.3 | 1,258 | 8 | 4.9 | <0.1 | 61,161 | 2,844 | 43.3 | 4.1 |  |
