# Supplementary material for: Comparative study on three viral enrichment approaches based on RNA extraction for plant virus/viroid detection using high-throughput sequencing
Source: PLoS One. 2020 Aug 25;15(8):e0237951. doi: 10.1371/journal.pone.0237951 (PMC7447037; doi:10.1371/journal.pone.0237951)
Supplement: S3 Table — Costs presented in Euros. (DOCX) [file pone.0237951.s004.docx]

**S3 Table. Average costs per sample of the three approaches used in the study.** Costs presented in Euros.

| **Approach** | **dsRNA** | | **Ribo-depleted totRNA** | | **sRNA** | |
| --- | --- | --- | --- | --- | --- | --- |
|  | **Method** | **Cost** | **Method** | **Cost** | **Method** | **Cost** |
| **Extraction kit** | Double-RNA Viral dsRNA Extraction Mini Kit for Plant Tissue (iNtRON) | 6.8 | innuPREP RNA Mini Kit (Analytik Jena AG) | 3.5‬ | innuPREP RNA Mini Kit (Analytik Jena AG) | 3.5‬ |
| **Enrichment** | NA | 0 | Ribodepletion | 77 | sRNA gel extraction | 535.7 |
| **Kit** | NA |  | RiboMinus Plant kit (Invitrogen) |  | Fastaris |  |
| **cDNA** | ProtoScript II First Strand cDNA Synthesis Kit | 4.5 | ProtoScript II First Strand cDNA Synthesis Kit | 4.5 | NA |  |
| **dscDNA** | NEBNext Ultra II Non-Directional RNA Second Strand Synthesis Module kit (NEB) | 15.5 | NEBNext Ultra II Non-Directional RNA Second Strand Synthesis Module kit (NEB) | 15.5 | NA |  |
| **Library** | Nextera XT Library Kit (Illumina)/Eurofins GATC | 280 | Nextera XT Library Kit (Illumina) | 280 | TruSeq small RNA kit (Illumina) |  |
| **Sequencing platform** | MiSeq (2x300 bp),  No. of reads 2,000,000 |  | MiSeq (2x300 bp),  No. of reads 2,000,000 |  | NextSeq 500 (1x50 bp), No. of reads 50,000,000 |  |
| **Total** | **306.9** | | **380.5** | | **539.2** | |
