# Supplementary material for: Comparative study on three viral enrichment approaches based on RNA extraction for plant virus/viroid detection using high-throughput sequencing
Source: PLoS One. 2020 Aug 25;15(8):e0237951. doi: 10.1371/journal.pone.0237951 (PMC7447037; doi:10.1371/journal.pone.0237951)
Supplement: S4 Table — (DOCX) [file pone.0237951.s005.docx]

**S4 Table. Pairwise comparisons of the nucleotide (nt) sequences and the amino acid (aa) sequence identities of Fr HZ11-065 isolates of PEMV1 and PEMV2 proteins with their most similar known isolates**

| **PEMV**  **Fr HZ11-065** | **Nucleotides** | | **Proteins** | | | |
| --- | --- | --- | --- | --- | --- | --- |
|  | **Identity** | **Reference** | **ORF** | | **Identity** | **Reference** |
| **PEMV1** | 95.7% | HM439775 | ORF1 | hypothetical 34 kDa protein | 96.0% | ADO86938 |
|  |  |  | ORF2 | hypothetical protein | 96.1% | ADO86939 |
|  |  |  | ORF3 | RNA-dependent RNA polymerase | 96.4% | ADO86940 |
|  |  |  | ORF4 | coat protein | 99.5% | ADO86941 |
|  |  |  | ORF5 | aphid transmission protein | 97.6% | ADO86942 |
| **PEMV2** | 93.5% | AY714213 | ORF1 | hypothetical protein | 92.5% | ALP43778 |
|  |  |  | ORF2 | RNA-dependent RNA polymerase | 95.6% | AAU20330 |
|  |  |  | ORF3 | phloem RNA movement protein | 96.5% | AAU20331 |
|  |  |  | ORF4 | cell-to-cell RNA movement protein | 97.6% | AAU20332 |
