# Supplementary material for: Comparative study on three viral enrichment approaches based on RNA extraction for plant virus/viroid detection using high-throughput sequencing
Source: PLoS One. 2020 Aug 25;15(8):e0237951. doi: 10.1371/journal.pone.0237951 (PMC7447037; doi:10.1371/journal.pone.0237951)
Supplement: S5 Table — (DOCX) [file pone.0237951.s006.docx]

**S5 Table. Pairwise comparisons of the four segments of the French isolate of WHAV2 (Fr HZ11-065) based on the nucleotide (nt) sequences and the amino acid (aa) sequences of their predicted proteins**

| **WHAV2**  **Fr HZ11-065** | **Nucleotides** | | **Proteins** | | | |
| --- | --- | --- | --- | --- | --- | --- |
|  | **Identity** | **Reference** | **ORF** | | **Identity** | **Reference** |
| **Segment 1** | 86.4% | NC_028382 | ORF1 | NS5-like | 96.8% | YP_009179378 |
| **Segment 2** | 90.7% | NC_028386 | ORF1 | VP4 | 96.9% | YP_009179384 |
|  |  |  | ORF2 | VP1 | 90.2% | YP_009179385 |
| **Segment 3** | 85.2% | NC_028383 | ORF1 | NS3-like | 95.5% | YP_009179379 |
| **Segment 4** | 85.6% | NC_028387 | ORF1 | VP2 | 92.0% | YP_009179386 |
|  |  |  | ORF2 | VP3 | 96.6% | YP_009179387 |
